# A Hybrid Electrospun-Extruded Polydioxanone Suture for Tendon Tissue Regeneration

**DOI:** 10.1089/ten.tea.2023.0273

**Published:** 2024-03-15

**Authors:** Roxanna E. Abhari, Sarah J.B. Snelling, Edyta Augustynak, Simon Davis, Roman Fischer, Andrew J. Carr, Pierre-Alexis Mouthuy

**Affiliations:** ^1^ Nuffield Department of Orthopaedics, Rheumatology, and Musculoskeletal Sciences, University of Oxford, Oxford, United Kingdom.; ^2^ Nuffield Department of Medicine, Target Discovery Institute, Centre for Medicines Discovery, University of Oxford, Oxford, United Kingdom.; ^3^ Nuffield Department of Medicine, Chinese Academy for Medical Sciences Oxford Institute, University of Oxford, Oxford, United Kingdom.

**Keywords:** tissue engineering, biomaterials, polymeric scaffolds

## Abstract

Many surgical tendon repairs fail despite advances in surgical materials and techniques. Tendon repair failure can be partially attributed to the tendon's poor intrinsic healing capacity and the repurposing of sutures from other clinical applications. Electrospun materials show promise as a biological scaffold to support endogenous tendon repair, but their relatively low tensile strength has limited their clinical translation. It is hypothesized that combining electrospun fibers with a material with increased tensile strength may improve the suture's mechanical properties while retaining biophysical cues necessary to encourage cell-mediated repair. This article describes the production of a hybrid electrospun-extruded suture with a sheath of submicron electrospun fibers and a core of melt-extruded fibers. The porosity and tensile strength of this hybrid suture is compared with an electrospun-only braided suture and clinically used sutures Vicryl and polydioxanone (PDS). Bioactivity is assessed by measuring the adsorbed serum proteins on electrospun and melt-extruded filaments using mass spectrometry. Human hamstring tendon fibroblast attachment and proliferation were quantified and compared between the hybrid and control sutures. Combining an electrospun sheath with melt-extruded cores created a hybrid braid with increased tensile strength (70.1 ± 0.3N) compared with an electrospun only suture (12.9 ± 1 N, *p* < 0.0001). The hybrid suture had a similar force at break to clinical sutures, but lower stiffness and stress. The Young's modulus was 772.6 ± 32 MPa for the hybrid suture, 1693.0 ± 69 MPa for PDS, and 3838.0 ± 132 MPa for Vicryl, *p* < 0.0001. Hybrid sutures had lower overall porosity than electrospun-only sutures (40 ± 4% and 60 ± 7%, respectively, *p* = 0.0018) but had a significantly larger overall porosity and average pore diameter compared with surgical sutures. There were similar clusters of adsorbed proteins on electrospun and melt-extruded filaments, which were distinct from PDS. Tendon fibroblast attachment and cell proliferation on hybrid and electrospun sutures were significantly higher than on clinical sutures. This study demonstrated that a bioactive suture with increased tensile strength and lower stiffness could be produced by adding a core of 10 μm melt-extruded fibers to a sheath of electrospun fibers. In contrast to currently used sutures, the hybrid sutures promoted a bioactive response: serum proteins adsorbed, and fibroblasts attached, survived, grew along the sutures, and adopted appropriate morphologies.

**Impact statement:**

This article describes the production and characterization of a bioactive suture with a sheath of submicron electrospun fibers and a core of melt-extruded fibers. The hybrid suture had a similar force at break to currently used surgical sutures, but increased porosity and a lower stiffness. In contrast to currently used sutures, hybrid sutures promoted a bioactive response: serum proteins adsorbed, and fibroblasts attached, proliferated, and adopted appropriate morphologies. These findings suggest that a hybrid electrospun-extruded suture has the potential to offer a similar bioactive advantage as electrospun-only devices, while having more comparable mechanical properties to currently used surgical sutures.

## Introduction

Many surgical tendon repairs do not heal successfully.^1^ Synthetic absorbable sutures are often used to connect the tendon to bone, or the tendon to tendon, depending on the type and location of the tear.^2^ There is both *in vivo* and observational evidence that the suture–tendon interface represents the weakest link in the repair and could be a driver for some repair failures.^3–7^ This is likely due to the tendon's poor intrinsic hypovascularity and hypocellularity, and the fact that sutures have been repurposed from other tissues and clinical uses and are not developed for the tendon's unique needs.^8–11^

Because the suture is the main foreign material in contact with torn tendon during surgical repair, it is a promising medium through which novel treatments can be explored. One possibility to improve tendon healing is by encouraging the repair of existing native tendon tissue through provision of biophysical cues, while providing mechanical support during the early repair process. Electrospun yarns could be used as bioactive sutures that employ biomimicry on two levels: through its submicron fibers mimicking tendon fibrils and through its hierarchical architecture similar to that of native ECM.^10–14^

There has been previous work in developing methods for producing continuous polydioxanone (PDO) electrospun filaments made up of submicron and nanoscale fibres.^15^ These filaments can be upscaled into braided multifilament yarns using textile machinery.^16^ Although biologically promising, the low tensile strength of electrospun materials compared with surgical sutures have limited the commercialization and clinical translation of many promising electrospun products.

It is hypothesized that combining electrospun fibers with a material with higher tensile strength may improve the suture's tensile strength while retaining biophysical cues, porosity, and surface area necessary to encourage cell-mediated repair.^10,17–19^ The purpose of this study is to develop a hybrid electrospun-extruded suture with a sheath of submicron electrospun fibers and a core of melt-extruded fibers of 10 μm. Current melt-extrusion technology is not capable of producing fibers that are <10 μm, offering fewer opportunities to mimic native ECM, in particular collagen fibrils, at the nano- and submicron scale.

However, microscale fibers offer other distinct advantages, such as their improved tensile strength, much faster production rate (on the order of 150–200 times faster), as well as being a continuous production process with fewer manual steps. Finally, there is evidence that native cells can attach to, and the degree of attachment, proliferation, and orientation be influenced by fibers up to 10 μm.^11,20^

This article describes the production of a hybrid electrospun-extruded suture with a sheath of submicron electrospun fibers and a core of melt-extruded fibres with 10 μm diameters. The properties of this suture are compared with three controls: an electrospun-only braided suture and two clinically used sutures, Vicryl and polydioxanone (PDS). Porosity, tensile strength, and bioactivity of the hybrid suture is assessed.

Bioactivity is assessed by measuring the adsorbed serum proteins on the raw electrospun and melt-extruded filaments using mass spectrometry, and quantifying tendon fibroblast attachment and proliferation on the hybrid suture, compared with on the control sutures.

## Materials and Methods

### Production of electrospun and melt-extruded filaments

The production of electrospun and melt-extruded filaments is described in detail in the Supplementary Data S1. In brief, a solution of 7% PDO with pyridine was used to fabricate continuous electrospun filaments using a custom apparatus. Melt-extruded filaments were fabricated from PDO using a Melt-Extrusion Spinning Line (Fibre Extrusion Technologies, Leeds, United Kingdom). Both types of filaments were drawn and annealed. The mass spectrometry proteomics data have been deposited to ProteomeXchange Consortium via the PRIDE partner repository. Accession number: PXD045312 (username: reviewer_pxd045312@ebi.ac.uk, password: 9oLfIToG).

### Braided hybrid suture production

Braided sutures were made using a braiding machine (Variation Braiding Machine; VF 1/[4–32]-140, Herzog Braiding Machine, Oldenburg, Germany). This industrial braiding machine had manually changeable crossings, enabling the production of braids using 4–32 carriers.^16^ A hybrid suture prototype was fabricated, made up of a 12 filament electrospun sheath with a core of 4 bundles of melt-extruded filaments (each filament had a 10 μm diameter). To do this, the braiding machine was set up with 12 fine yarn carriers with electrospun filaments. In addition, four fine yarn carriers were positioned under the braiding machine, so that four bundles of melt-extruded filaments could be fed through the center of the electrospun filament carriers continuously as the cores. A braided suture made up of 12 electrospun filaments (without cores) was fabricated as a control.

### Material characterization using scanning electron microscopy

An optical micrometer (Keyence LS-7010MR laser with Keyence LS-7601 monitor, Milton Keynes, United Kingdom) was used to measure suture diameter by averaging five measurements, which was then converted to United States Pharmacopeia (USP) suture sizing standardization. Scanning electron microscopy (SEM) was used to measure fiber diameter. Suture materials measuring 2–3 cm were cut and mounted on aluminum stubs (Agar Scientific, Essex, United Kingdom) using carbon tape (Agar Scientific).

These stubs were coated with gold for 120 s using a SC7620 Mini Sputter Coater System (Quorum Technologies Ltd, Laughton, United Kingdom). SEM images were acquired using an EVO LS15 VP-SEM (Carl Zeiss, EVO LS15, Oberkochen, Germany) in high vacuum mode to examine suture morphology. Fiber diameter was measured from SEM images using ImageJ software (National Institutes of Health, Bethesda, MD). For each experimental condition, 3 samples were imaged and 10 measurements per image were captured at magnifications between 200 × and 1000 × .

### Porosity assessed by Hg porosimetry

Average pore diameter and overall porosity was determined using an Autopore IV 9500 mercury porosimeter (Micromeritics Instrument Co, Norcross, GA). The porosimeter had a maximum pressure range of 2.27 × 10^8^ Pascal psia and a measurable pore size range of 360–0.005 μm. Braided sutures were cut into sections weighing 70–100 mg and placed in the penetrometer fixed with a sample cup bonded to a metal-clad glass-capillary stem (s/n = 14, 3 bulb, 0.412 stem, powder).

The penetrometer was first passed through the low-pressure port where the gases were evacuated from the penetrometer and then backfilled with mercury. Then, the penetrometer was passed through the high-pressure port, where pressure on the penetrometer increased and mercury intruded into the sample pores, beginning with the pores of the largest diameter. Samples were run with a mercury filing pressure of 0.49 psia and an equilibration time of 10 s. Two experimental repeats were performed for the hybrid and control sutures.

### Tensile testing and cross-sectional area measurement by microcomputed tomography

Cross-sectional area (CSA) measurements were made using microcomputed tomography (μCT), described in detail in the Supplementary Data S1. The CSA calculations for stress measurements were obtained by multiplying the μCT solid CSA by the percentage of solid area obtained from Hg porosimetry. Braided sutures were cut into 20 cm sections and uniaxial tensile testing to failure was performed at a crosshead speed of 50 mm/min with a 5 kN load cell until failure using a Zwick tensile testing machine (Zwick Roell Group, USA) equipped with the custom grips that have been previously described.^16^ Force at break (*N*), ultimate stress (MPa), ultimate breaking strain (%), and Young's modulus (MPa) were recorded. Five experimental repeats were performed for hybrid and control sutures.

For all quantitative analyses, data are presented as mean ± standard deviation. For statistical comparison, a one-way analysis of variance followed by a Tukey *post hoc* test was performed to identify significance between each group. GraphPad PRISM version 9 software (GraphPad Software Inc., La Jolla, CA) was used for all statistical analysis.

### Serum protein adsorption using mass spectrometry

Sutures were mounted on cell crowns and soaked in 200 μL of healthy human serum (*n* = 4, 2 males, 2 females; average age 27) followed by incubation for 1 h at 37°C. Bound protein was removed and digested from the washed scaffolds using 500 ng trypsin (Promega, Southampton, United Kingdom) in 50 mM ammonium bicarbonate at 37°C overnight. Sample preparation is described in detail in Supplementary Data S1.

Samples were then processed by research staff at the Target Discovery Institute (University of Oxford, United Kingdom) using adapted in-house protocols. Proteins were digested directly from washed materials by the addition of 1 μg of trypsin (Promega) in 50 mM ammonium bicarbonate overnight at 37°C. The resulting peptides were purified using SOLA C18 HRP (Thermo) desalting cartridges. Eluted peptides were dried, resuspended in 2% acetonitrile, 0.1% trifluoroacetic acid, and stored at −20°C before analysis.

Peptides were detected by nano-UPLC-MS/MS over a 60-min gradient of 0.1% formic acid in 5% dimethyl sulfoxide (DMSO) to 0.1% formic acid to 35% acetonitrile in 5% DMSO using a Dionex Ultimate 3000 nano-UPLC with an EASY-Spray column (75 × 500 μm, 2 μm particle size, ThermoFisher Scientific, Waltham, MA) coupled to a Q-Exactive Mass Spectrometer (ThermoFisher Scientific). MS1 spectra were acquired between 375 and 1500 m/z with a resolution of 70,000. MS2 spectra of higher-energy collisional dissociation fragmented precursors were acquired with a resolution of 17,500. Selected precursors were excluded from repeated analysis for 27 s. Proteomic data analysis is described in the Supplementary Data S1.

### Tendon fibroblast attachment and metabolic activity

Tendon fibroblasts were isolated from biopsies of hamstring tendons from healthy donors. These were isolated, expanded, and seeded on hybrid sutures and controls. Cell culture methods are described in detail in Supplementary Data S1. Initial cell attachment was determined at 24 h after seeding. At the 24-h time point, sutures were carefully removed from the cell crowns and transferred into a new well containing 2 mL of a solution of 10% Prestoblue (Invitrogen, Paisley, United Kingdom) and 90% culture media and incubated for 2 h. A calibration curve was generated by seeding cells at the following densities in a 24-well plate (Corning Inc., Corning, NY): 5 × 10,^3^ 1 × 10,^4^ 2 × 10,^4^ 4 × 10,^4^ 6 × 10,^4^ 8 × 10,^4^ and 1 × 10.^5^

The calibration curve was used to approximate initial cell number attached, as represented by Prestoblue initial attachment measurements. After incubation, the fluorescence of each solution was measured in triplicates on a FluoStar Optima microplate reader (BMG Labtech, Ortenberg, Germany) with an excitation of 485 nm and an emission of 520 nm. Sutures were then maintained in culture media without crowns for the duration of the experiment.

Cell proliferation was assessed using a Prestoblue assay at days 4, 7, and 14 after seeding for hybrid and control sutures. A calibration curve was generated by seeding cells at the following densities in a 24-well plate (Corning Inc.): 5 × 10,^3^ 1 × 10,^4^ 2 × 10,^4^ 4 × 10,^4^ 6 × 10,^4^ 8 × 10,^4^ and 1 × 10.^5^ The calibration curve was used to approximate cell number as represented by Prestoblue metabolic activity measurements, throughout the experiment.

## Results

### Hybrid suture characteristics

**FIG. 1.**
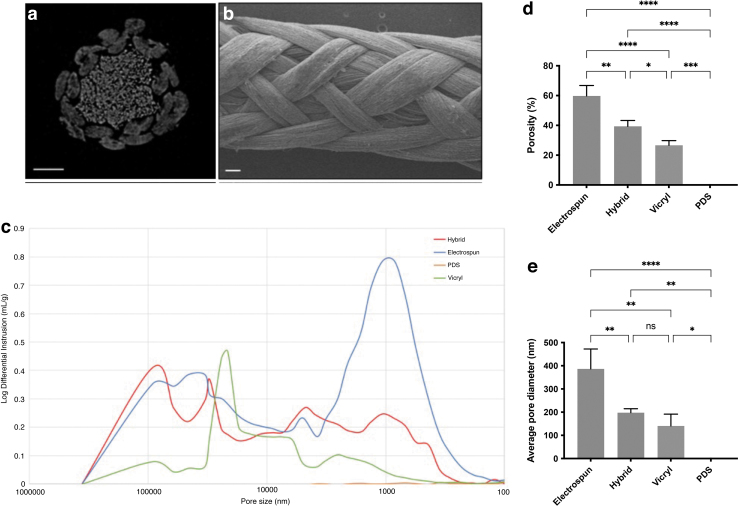
Material characterization of the hybrid electrospun-extruded suture using SEM and Hg porosimetry. **(a)** Cross-sectional μCT scan showing the 12 filament electrospun sheath with 4 melt-extruded cores. Scale bar 200 μm. **(b)** SEM image showing suture morphology with both electrospun and melt-extruded fibers visible on the surface. Scale bar 100 μm. **(c)** Representative pore size distribution of sutures measured by Hg porosimetry. The two most frequent pore diameters of the electrospun and hybrid suture were between 0.5 and 5 μm and between 50 and 100 μm. The Vicryl curve showed minimal intrusion, with a peak between 20 and 50 μm, and the PDS curve displayed no intrusion. **(d)** Porosity and average pore diameter of sutures using Hg porosimetry. Overall porosity (%) was significantly higher for the electrospun suture compared with the hybrid suture (*p* = 0.0018) and compared with Vicryl (*p* < 0.0001). **(e)** Average pore diameter (nm) was significantly higher for electrospun suture compared with the hybrid suture (*p* = 0.0079) and compared with Vicryl (*p* = 0.0015). Statistical significance was determined at *p < 0.05, **p < 0.01, ***p < 0.001, and ****p < 0.0001. Error bars represent standard deviations; *n* = 3 for all conditions. μCT, microcomputed tomography; NI, no intrusion; ns, not significant; PDS, polydioxanone; SEM, scanning electron microscopy.

Hybrid braided sutures were manufactured using 12 continuous electrospun filaments and 4 bundles of continuous melt-extruded filaments. The resulting braid diameter was 0.69 ± 0.02 mm (between USP size 2 and 3). The surface and cross-section of the braided suture can be seen using μCT and SEM, respectively (Fig. 1a, b). The extruded fibers are visible in the core of the suture and are also visible on the surface of the sutures.

The difference in porosity between the hybrid and control sutures were measured by Hg porosimetry (Fig. 1d, e). The overall porosity was 60 ± 7% for the electrospun suture, compared with 40 ± 4% for the hybrid suture (*p* = 0.0018) and 27 ± 3% for the Vicryl suture (*p* < 0.0001). The porosity of the hybrid suture was also higher than Vicryl (*p* = 0.028). The average pore diameter was 386 ± 86 nm for the electrospun suture compared with 198 ± 17 nm for the hybrid suture (*p* = 0.0079) and 141 ± 51 nm for the Vicryl suture (*p* = 0.0015).

The PDS suture displayed no intrusion. A representative intrusion curve comparing the pore size distribution between the hybrid and control sutures is presented in Figure 1c. There were two local maxima of the electrospun suture intrusion curve, which represents the most frequent pore diameter: one ∼0.5–5 μm and the other between 50 and 100 μm. The intrusion curve from the hybrid suture showed a similar range of pore sizes, but with less intrusion. The Vicryl curve showed minimal intrusion, with a peak between 20 and 50 μm, and the PDS curve displayed no intrusion.

### Hybrid sutures have a similar breaking force but a lower stiffness than clinical sutures

**FIG. 2.**
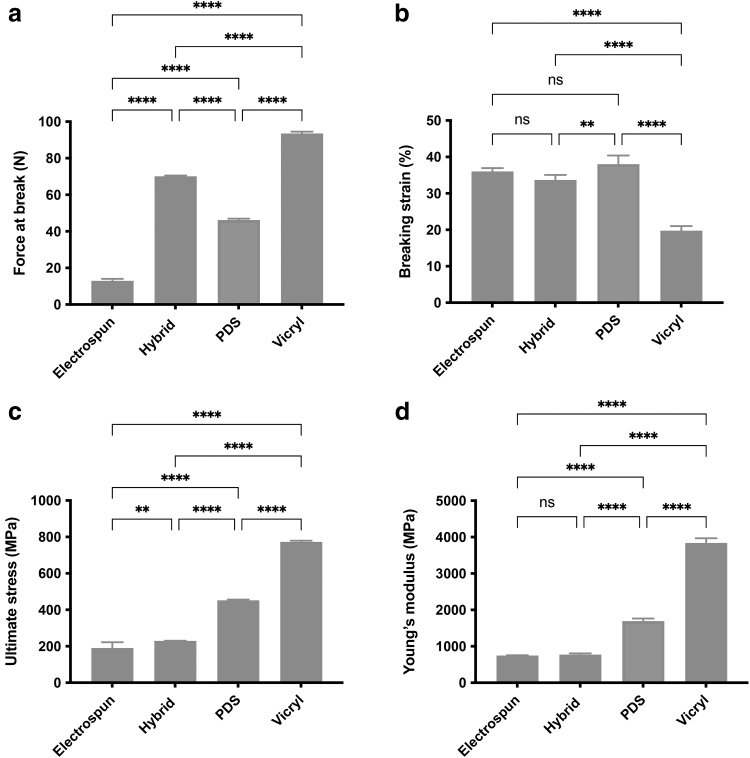
Mechanical testing of hybrid and control sutures. **(a–d)** Tensile properties of braided electrospun, hybrid, and control surgical sutures. Force at break (*N*) was lowest for the electrospun suture and highest for Vicryl, but all significantly different from each other (*p* < 0.0001); breaking strain (%) was lowest for Vicryl compared with all sutures (*p* < 0.0001); ultimate stress (MPa) of the electrospun suture was lower than the hybrid (*p* = 0.008), PDS (*p* < 0.0001), and Vicryl (*p* < 0.0001) sutures; Young's modulus (MPa) was lower for electrospun and hybrid compared with both surgical sutures (*p* < 0.0001). Statistical significance was determined at *p < 0.05, **p < 0.01, ***p < 0.001, and ****p < 0.0001. Error bars represent standard deviations; *n* = 5 for each condition.

There were significant differences in mechanical properties and behavior between hybrid and control sutures, as shown in Figure 2. A representative stress–strain graph is shown in Supplementary Figure SA1. All sutures failed in the mid-substance region and all multifilament sutures (hybrid, electrospun, and Vicryl) showed characteristic toe, linear, and yield regions. The force to failure was 12.9 ± 1 N for the electrospun suture, 70.1 ± 0.3 N for the hybrid suture, 46.2 ± 0.6 N for PDS, and 93.6 ± 0.8 N for Vicryl, were all significantly different from each other (*p* < 0.0001).

The breaking strain of Vicryl was 19.8 ± 1%, which was lower than that of the electrospun suture (36 ± 1%), the hybrid suture (33.7 ± 1%), and PDS (38 ± 2%), *p* < 0.0001. The average ultimate stress was 190.2 ± 32 MPa for the electrospun suture, which was lower than the hybrid suture (229.3 ± 1 MPa, *p* = 0.008), PDS (451.7 ± 4 MPa, *p* < 0.0001), and Vicryl (773.3 ± 6 MPa, *p* < 0.0001). These results were all statistically different from each other. The Young's modulus was 747.1 ± 8 MPa for the electrospun suture and 772.6 ± 32 MPa for the hybrid suture, which were lower than that of PDS (1693.0 ± 69 MPa) and of Vicryl (3838.0 ± 132 MPa), *p* < 0.0001.

### Similar serum proteins adsorbed to electrospun and melt-extruded filaments

**FIG. 3.**
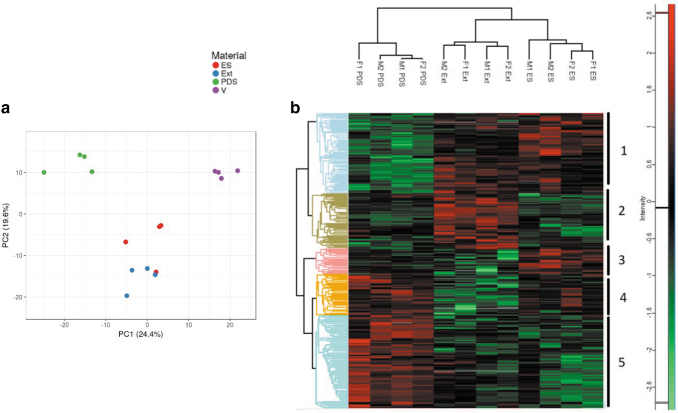
Protein corona composition of hybrid and control sutures. **(a)** Principal component analysis of protein coronas formed on electrospun filaments (Es) and melt-extruded yarns (Ext), as well as on surgical sutures PDS (PDS) and Vicryl (V). There is less separation between electrospun filaments and melt-extruded yarns than compared with, and between, PDS and Vicryl. **(b)** Heat map of differentially expressed proteins in the coronas of PDS sutures, electrospun filaments (Es), and melt-extruded yarns (Ext). These three materials were incubated in four serum samples (M1, M2, F1, and F2) for 1 h before mass spectrometry analysis of bound proteins. There were five main clusters of bound proteins detected, totaling 338 differentially expressed proteins. Cluster 1 increased in intensity with electrospun filaments and melt-extruded yarns; cluster 2 increased in intensity with extruded filaments only; cluster 3 increased with electrospun filaments only; cluster 4 increased with electrospun and PDS sutures; and finally, cluster 5 increased with PDS sutures only. There was little variation between the four serums tested. Log10 scale; *red* represents increasing intensity and *green* represents decreasing intensity.

The principal component analysis graph showed a clear separation between electrospun filaments and melt-extruded yarns, compared with PDS and Vicryl sutures (Fig. 3a). Vicryl has different physical and chemical properties compared with the other three materials and was excluded specific protein analysis better discern differences between PDO samples. Figure 3b shows a heat map of proteins that were expressed in the coronas of PDS, electrospun filaments, and melt-extruded yarns. Overall, there were 338 proteins that were statistically significantly different from each other.

These proteins formed five main clusters, as shown in Figure 3b and Supplementary Figure SA2. The most significant differences were seen in the PDS suture corona compared with that of the other two materials. Clusters 1 and 5 represent the largest groups of proteins that were differentially expressed (105 and 126 proteins, respectively). Cluster 1 was enriched with electrospun filaments and extruded yarns, whereas cluster 5 was enriched with PDS. Both clusters 1 and 5 were driven by binding proteins and catalytic activity proteins, and this represents the largest group of proteins across all groups.

Understanding the function of the proteins differentially expressed in these groups has implications for understanding what drives the initial cell attachment and immune cell response on the hybrid suture and on PDS. Supplementary Figure SA3 compares the abundance of binding proteins between the materials tested with a focus on binding proteins that are deemed especially important for fibroblast attachment. Proteins such as fibronectin, vitronectin, and laminin, which are thought to be important in mediating cell attachment, were more highly abundant on electrospun and melt-extruded filaments, compared with on PDS.

### Increased fibroblast attachment and proliferation on hybrid sutures compared to on clinical sutures

**FIG. 4.**
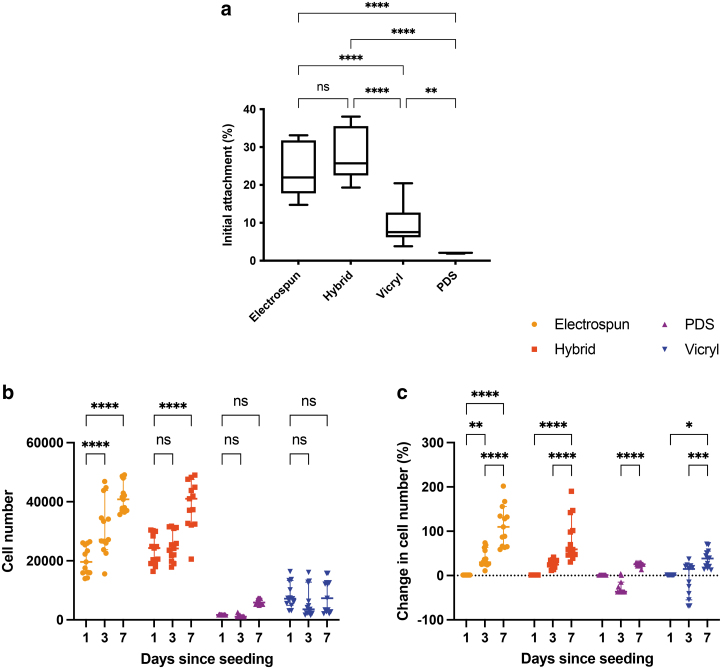
Tendon fibroblast attachment and metabolic activity on hybrid and control sutures. **(a)** Initial attachment was higher on hybrid (28 ± 7%) and electrospun sutures (24 ± 7%), compared with on Vicryl (10 ± 5%) and PDS (2 ± 0.1%), *p* < 0.0001. Vicryl also had a significantly higher initial attachment compared with PDS (*p* = 0.002). **(b)** Cell number on hybrid and control sutures was compared on days 1, 3, and 7. On days 3 and 7, there were more cells attached to the hybrid and electrospun suture than to PDS and Vicryl (*p* < 0.0001). There was no significant difference between electrospun and hybrid sutures or between Vicryl and PDS sutures on days 3 or 7. **(c)** For each material, the change in cell number was measured relative to day 1. On day 3, there was an increase in cell number on hybrid sutures (*p* = 0.0247) and electrospun sutures (*p* = 0.0003) and a decrease on PDS sutures (*p* = 0.0082). On day 7, there was an increase in cell number on hybrid and electrospun sutures (*p* < 0.0001 for both), PDS sutures (*p* = 0.0364), and Vicryl sutures (*p* = 0.0007). Statistical significance was determined at *p < 0.05, **p < 0.01, ***p < 0.001, and ****p < 0.0001; Error bars represent standard deviations; *n* = 3 for each condition.

The initial attachment of tendon fibroblasts on hybrid sutures compared with on control sutures is shown in Figure 4a. At 24 h after seeding, there was 28 ± 7% attachment to hybrid sutures, 24 ± 7% to electrospun sutures, 10 ± 5% to Vicryl, and 2 ± 0.1% to PDS. The attachment on electrospun and hybrid sutures was significantly higher than on Vicryl and PDS, *p* < 0.0001. There was also a significantly higher attachment on Vicryl compared with on PDS, *p* = 0.002.

Figure 4b compares cell number measured on hybrid and control sutures over 7 days. On days 3 and 7, the cell number was higher for hybrid and electrospun sutures, compared with on Vicryl and PDS (*p* < 0.0001 for all comparisons). Figure 4c shows the change in cell number measured relative to day 1 for each material. On day 3, there was an increase in cell number on hybrid sutures (*p* = 0.0247) and electrospun sutures (*p* = 0.0003), and a decrease on PDS sutures (*p* = 0.0082). On day 7, there was an increase in cell number on hybrid and electrospun sutures (*p* < 0.0001 for both), PDS sutures (*p* = 0.0364), and Vicryl sutures (*p* = 0.0007).

**FIG. 5.**
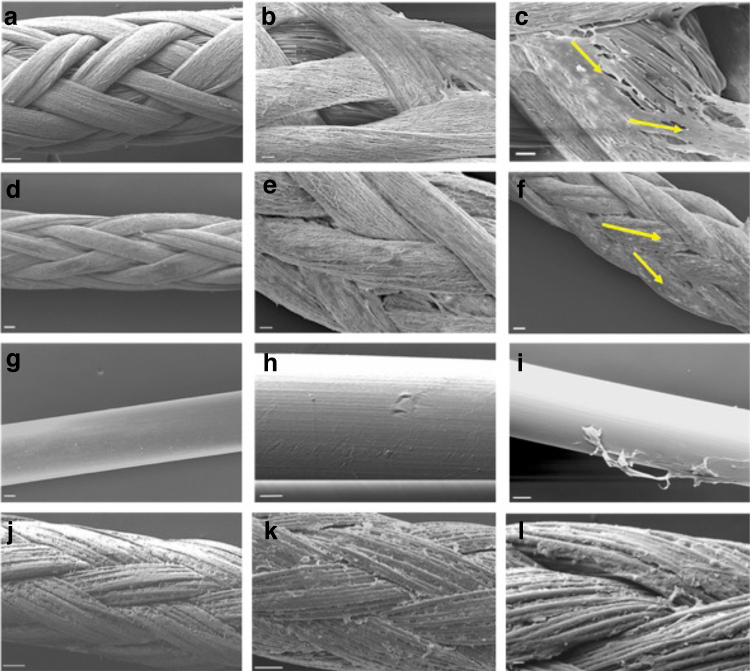
SEM images of healthy tendon fibroblasts seeded on hybrid and control sutures. The *left column* shows the unseeded sutures; the *middle* column shows the sutures cultured for 4 days; the *right column* shows the cells cultured for 7 days. **(a–c)** Hybrid sutures. **(a)** Hybrid sutures without cells. **(b)** Cells show preferential attachment to electrospun over melt-extruded fibers at 4 days. **(c)** Cells are growing and migrating to attach to the melt-extruded fibers at 7 days; however, their morphology is not as clear as on electrospun fibers. **(d–f)** Electrospun control suture. **(d)** Electrospun suture without cells. **(e)** Cell attachment to electrospun fibers with individual cells visible. **(f)** Significant cell attachment and elongation (shown with *yellow arrows*) along fibers at 7 days. **(g–i)** PDS control suture. **(g)** PDS sutures without cells. **(h)** No visible cells at 4 days. **(i)** Some marking on the suture at 7 days, but unclear if these are cells. **(j–l)** Vicryl control suture. **(j)** Vicryl sutures without cells. **(k)** Some cells potentially attached at 4 days, but difficult to distinguish from the suture coating. **(l)** Some potential cell coverage at 7 days, with cells exhibiting a rounded morphology. Scale bars are 50 μm for **(a–e** and **g–k** and 20 μm for **f** and **i)**.

There were differences in cell coverage on hybrid sutures compared with on control sutures. Figure 5 shows the four suture types without cells (left column), cultured with tendon fibroblasts with 4 days (middle column), and cultured for 7 days (right column). On day 4, there was cell coverage on the hybrid suture, but it was localized to the electrospun component (Fig. 5b). Conversely, there was minimal cell coverage on PDS (Fig. 5h) or Vicryl (Fig. 5k).

On day 7, tendon fibroblasts elongated along electrospun filaments resembling the morphology seen in healthy human tendons (Fig. 5c, f). There was some attachment of tendon fibroblasts on melt-extruded fibers in the hybrid suture, as shown in Figure 5f, but there was visibly reduced coverage on these fibers compared with on electrospun sutures (Fig. 5c). Overall, fibroblasts seemed to conform to fiber geometry on electrospun sutures, but not as much on hybrid sutures.

Cells did not visibly attach on PDS sutures (Fig. 5I). Owing to the cells forming confluent aggregates, it was difficult to discern cell shape. There was some attachment on Vicryl sutures; however, these cells took on a more textured and rounded shape with no preferential direction (Fig. 5l). Because of the coating on Vicryl sutures, it was more difficult to discern cell shape (Fig. 5k, l).

## Discussion

The objective of this article was to develop a hybrid electrospun-extruded suture, with comparable mechanical properties to clinically used sutures Vicryl and PDS, but with improved bioactivity, assessed by the composition of the adsorbed protein corona and fibroblast–material interaction. In support of our hypotheses, combining an electrospun sheath with melt-extruded cores created a hybrid braid with increased tensile strength.

Compared with surgical sutures, the hybrid sutures had a similar force at break, but were more porous and had a lower stiffness and stress. Similar protein compositions were present on electrospun and melt-extruded filaments, and the hybrid suture supported more tendon fibroblast attachment and proliferation compared with on clinical sutures.

A popular tissue engineering design hypothesis is that a regenerative device should balance mechanical strength with porosity to allow for cellular infiltration, which can present conflicting design requirements.^21–23^ In this study, Hg porosimetry revealed that the hybrid and electrospun suture had a higher overall porosity and larger pore sizes compared with the clinical sutures. The hybrid suture had a lower overall porosity and pore diameter compared with the electrospun suture; it is possible that braiding around a core created a tighter structure with less pronounced intrusion.

The Vicryl intrusion graph showed some interstitial filling with one peak ∼20–50 μm, indicating minimal space between the braided bundles of fibers. As anticipated, the monofilament PDS intrusion graph showed no intrusion. Porosity of nano- and submicron fibers is notoriously difficult to study as there are no clear targets as to how porous a suture should be, and how the requirements of the tissue will change over time. It is also difficult to study in a static environment, as the pore shapes and sizes within the structure would likely change under mechanical loading.

Tensile testing revealed that the electrospun and hybrid sutures had different mechanical properties compared with PDS and Vicryl. Compared with the clinical sutures, the force at failure of electrospun sutures was three times lower than PDS and seven times lower than Vicryl. The differences in ultimate stress and Young's modulus between the hybrid and electrospun sutures compared with PDS is possibly due to variations in surface area. The electrospun and hybrid sutures are made up of a higher density of fibers with smaller diameters, leading to a much larger overall surface area. None of the sutures matched the force at failure of native tissue.

Previous work measured the maximum force at failure of supraspinatus tendon in a cadaveric specimen to be 311 ± 41 N, with previous reported values ranging between 80 and 652 N.^24,25^ It is not clear what the physiological range of the supraspinatus tendon is, although it will vary between healthy and diseased tendon. Previous work measured the Young's modulus of supraspinatus tendon to be between 1000 and 2000 MPa,^26^ which is close to the stiffness of the electrospun, hybrid, and PDS sutures (all <2000 MPa), but is much lower than that of the Vicryl suture (∼4000 MPa).

Although it is important for the sutures repairing a torn tendon to be stiff enough to effectively transfer forces, if it is too stiff, it could increase the chance of re-tearing the tendon by causing pull through. Moreover, the mechanical properties required of a suture to augment tendon repair will change over time, as native tissue begins to replace the degraded suture. However, it is not clear how stiff the suture should be and how that stiffness should change over its lifetime, to promote the optimal repair. Future work characterizing both the macro- and micromechanical properties of healthy and diseased tendon would help inform better matching of surgical materials to the requirements of the tissue.

Cell–material interactions are mediated by an adsorbed protein layer, which is formed on the material surface upon initial contact with serum and precedes the attachment of immune and stromal cells.^27,28^ It is through this adsorbed layer that tendon fibroblasts and immune cells initially respond.^29,30^ Electrospun and extruded filaments had more similar adsorption profiles compared with Vicryl and PDS. Fibronectin, vitronectin, and laminin, thought to be important proteins in mediating fibroblast attachment, were more highly abundant on electrospun and melt-extruded filaments, compared with on PDS.

This finding also correlates with a higher initial fibroblast attachment on electrospun-based sutures, compared with on PDS. The type, abundance, and orientation of proteins is largely dependent on chemical and physical properties of the material and influences how cells interact with the material.^31^ In terms of gross chemical differences, electrospun filaments, melt-extruded yarns, and PDS sutures are all made of PDO, whereas Vicryl is made of poly-l-lactate. In considering physical differences, electrospun and melt-extruded filaments are made up of 1 and 10 μm sized fibers, respectively, whereas PDS and Vicryl are made up of larger fibers (on the order of 3–30 times larger).

Although not directly assessed here, local surface properties, such as surface hydrophobicity,^32,33^ charge,^33^ and chemistry,^34^ have been shown to be key determinants in altering protein binding and fibroblast behaviour.^27,34,35^ Indeed, tendon fibroblast attachment and proliferation varied between the different sutures. At 24 h after seeding, there was similar initial cell attachment on hybrid and electrospun sutures. This result was expected, as previous studies support the idea that the submicron size of electrospun fibers supports cellular adhesion.^36–38^

Electrospun sutures promoted the largest increase in cell number over a 14-day culture period. This result is supported by previous studies, which suggested that an increase in surface area available for cellular attachment is not always correlated with increased cell proliferation.^39,40^ It might be that electrospun fibers, or the proteins attached to them, have more specific cues that drive proliferation. Future work should interrogate the proteins differentially expressed on the electrospun-based sutures, to investigate which attached proteins are most likely to promote fibroblast attachment and proliferation.

In contrast, very few cells attached to the surgical sutures, which is consistent with previous *in vitro* and *in vivo* experiments.^41–43^ Wong et al. investigated the cellular activity of the tendon–suture interface in a rabbit and mouse model and found a well-demarcated acellular zone around the Prolene suture (a nonabsorbable synthetic suture) as soon as 24 h after implantation.^42^ A similar acellular zone was observed by Rashid et al., between tendon tissue and a PDS suture in an ovine model.^44^ Although proteins did adsorb to the PDS and Vicryl surface, these results suggest that they might not be the ones providing cues for attachment.

There are several strengths and limitations of this study. To our knowledge, this was the first study to combine electrospun and melt-extruded filaments to fabricate a core-sheath suture. Previous studies have explored electrospinning as a method to develop core-sheath monofilaments, most notably for use as sacrificial fibers or drug eluting sutures.^45–48^ The potential for the core and sheath to have different properties, as was shown here, demonstrates the breadth of potential clinical applications for this hybrid device.

Moreover, this was the first study to directly compare the porosity, tensile strength, and bioactivity of electrospun-based sutures to those of commercially used clinical sutures. A limitation of this study is that fibroblasts from healthy tissue was used and only one cell type was studied. Previous work investigating the response of both healthy and diseased tendon fibroblasts to electrospun fibers showed that diseased cells retain the ability that healthy cells have in responding to biophysical cues, although at a different rate.^49,50^

Future study should consider how the immune signature is influenced by the adsorbed protein corona and how immune cells regulate the recruitment of fibroblasts.^51,52^ A further limitation is that no power analysis was performed to determine the sample size for tensile testing, and that differences in surface material properties were not compared. Future study should further explore surface properties, using X-ray photoelectron spectroscopy and atomic force microscopy, to characterize chemical elements at the surface and how they influence protein adsorption.

## Conclusion

This article describes the production and characterization of a hybrid electrospun-extruded suture with potential for augmenting the repair of torn tendon. Adding melt-extruded cores to an electrospun sheath increased the suture's tensile strength, a commonly limiting factor in the clinical translation of electrospun materials. The hybrid suture had a similar force at break to surgical sutures, but increased porosity and a lower stiffness.

In contrast to currently used sutures, the hybrid sutures promoted a bioactive response: serum proteins adsorbed, and fibroblasts attached, survived, grew along the sutures, and adopted appropriate morphologies. Future study should assess the local material properties and evaluate fibroblast response in conjunction with other immune and stromal cells to further elucidate its bioactive potential.

## Supplementary Material

Supplemental data

Supplemental data

Supplemental data

Supplemental data

## Supplementary Material

Supplementary Data S1

Supplementary Figure SA1

Supplementary Figure SA2

Supplementary Figure SA3
