# Supplementary material for: A Hybrid Electrospun-Extruded Polydioxanone Suture for Tendon Tissue Regeneration
Source: Tissue Eng Part A. 2024 Mar 15;30(5-6):214–24. doi: 10.1089/ten.tea.2023.0273 (PMC10954604; doi:10.1089/ten.tea.2023.0273)
Supplement: Supplemental data [file Suppl_DataS1.docx]

**Supplementary methods**

Production of electrospun filaments

A custom electrospinning apparatus with a single nozzle and stainless-steel wire collector was used to fabricate continuous electrospun filaments (17). Polydioxanone (PDO; Riverpoint Medical, Portland, USA) was dissolved in 1,1,1,3,3,3-hexafluoroisopropanol (HFIP; Halocarbon Product Corporation, Atlanta, USA) at a concentration of 7% (w/v) in the presence of pyridine. A 5mL syringe pump (Harvard Apparatus, Kent, UK) delivered PDO solution at a feed rate of 0.8mL/hour. The wire feed rate was 0.5mm/second and the filament was spun under an electric field of 6.5-9.5kV. The filaments were detached from the collecting wire and were manually stretched until resistance was felt. The stretched filaments were then thermally annealed at 65°C for 3 hours as previously described and stored at 25°C in a desiccator (23).

Production of melt-extruded filaments

Melt-extruded filaments were produced with a Melt-Extrusion Spinning Line (Fibre Extrusion Technologies, Leeds, UK) using a 20mm diameter extruder and a 1mL/rev melt pump. PDO was supplied by Riverpoint Medical (Portland, OR, USA) in individual lots of 100g characterised by melt flow rate (MFR), a surrogate measurement to molecular weight. Two lots of PDO with MFRs of 3.9g/10min and 4.5g/10min at 170°C were blended at a 1:1 ratio to yield a final blend of 400g. Once blended, the resin was transferred to the feed of the extruder and heated. Molten PDO was then passed through a metal spinneret with 28 holes of 0.2mm in diameter. The fibres were drawn at a ratio of 4:1, initially in the melt and then the solid phase, over a series of paired ceramic rollers to yield a bundle of fibres. Each bundle was made up of 28 fibres with a 10μm diameter. To produce a stable final filament, bundles were combined using a ring twister, first combining 4 bundles in one direction (S twist) and then combining 3 twisted bundles in the other direction (Z twist). This yielded a final twist-neutral filament with a diameter of 278μm, made up of 12 filaments.

Cross-sectional area measurement by μCT

Cross-sectional area measurements were made using micro-computed tomography (μCT). Hybrid sutures were scanned over a length of 1mm. The sutures were placed in a tube that was mounted vertically on the μCT scanner (SkyScan 1172, SkyScan, Kontich, Belgium). The samples were scanned at an isotropic pixel size between 1-3μm. The scan parameters were set at a voltage of 40 kV; a current of 250 mA without filter and 900 projections were used. The images were reconstructed using NRecon software (SkyScan 1172, SkyScan, Kontich, Belgium) using the Feldkamp algorithm with a beam-hardening correction of 40% and a smoothing of 4. CTAn software version 1.12 (Bruker microCT, Kontich, Belgium) was used for processing and stacking the reconstructions into a tubular structure. ImageJ software version 1.52a (National Institute of Health, Bethesda, MD, USA) was used to measure the suture’s CSA, diameter, and overall porosity.

Sample preparation for protein adsorption experiment

Material preparation

To characterise the composition of the adsorbed protein corona on hybrid sutures, electrospun sutures, and control sutures were mounted on cell crowns (24-well plate size, Scaffdex, Tampere, Finland). Cell crowns were sterilized for 2 hours in 70% ethanol, then washed 3 times with PBS and left to air dry overnight.

Material functionalisation with human serum

Serum was collected with appropriate ethical approval (REC11/H0711/7). Blood was collected from donors and left at room temperature to clot for 30 minutes. The blood samples were centrifuged at 1300g for 15 minutes, then the serum was lifted off and aliquoted to 500μL. Crowns (n=3 per filament or suture type) were soaked in 200μL of healthy human serum (n=4, 2 males, 2 females, average age 27) followed by incubation for 1 hour at 37°C.

Mass spectrometry of adsorbed protein corona

Following serum adsorption, the material was removed from crowns and placed in 2mL of PBS on a plate shaker with gentle agitation for 5 minutes. This washing step was repeated two more times to fully remove any serum or loosely bound protein. The material was then clasped in a Lo Bind Eppendorf tube (Eppendorf AG, Hamburg, Germany) before brief centrifugation to remove any remaining PBS and unbound protein from the material. Bound protein was removed and digested from the washed scaffolds using 500ng trypsin (Promega, Southampton, UK) in 50mM ammonium bicarbonate at 37°C overnight.

Sample preparation for tendon fibroblast experiment

Human tendon tissue acquisition

Biopsies of hamstring tendons were acquired from healthy donors with informed donor consent under ethics from the Oxford Musculoskeletal Biobank (REC 09/H0606/11) and in agreement with Institutional and National ethical requirements. During surgery, hamstring biopsies were taken from residual healthy tendon from patients undergoing ACL repair. These biopsies were transferred from the patient to sterile tubes containing culture media and transported to the laboratory for fibroblast isolation and expansion.

Tendon fibroblast isolation and expansion

Tendon fibroblasts were extracted by cutting the tissue biopsies into small pieces, approximately 2mm × 2mm before transferring to 6-well plates (Corning Inc., Corning, NY, USA). Explants were initially cultured in supplemented media made up of DMEM/F12 media (Lonza, Slough, UK), supplemented with 50% Fetal Bovine Serum (FBS, Labtech, Lonza, Slough, UK) and 1% penicillin-streptomycin (P/S, ThermoFisher Scientific, Waltham, MA, USA). This media was replaced every three days with D10 media used once outgrowth from explants was observed. Once the cell had reached confluence, indicated by tendon fibroblasts migrating from the tendon onto the 6-well plate, they were scraped and transferred to 10cm Petri dishes (Greiner, Frickenhausen, Germany). Cells were expanded until 80-90% confluent and were used at passage 3 to avoid the phenotypic drift observed at higher passages(1).

Tendon fibroblast seeding and culture

For all tissue culture experiments, cells were seeded in 12-well plates at a density of 80,000 cells/crown in 200μL in a static environment. Two pieces of 4cm section of suture were mounted per cell crown (24-well plate size, Scaffdex, Tampere, Finland). Care was taken so that separate filaments weren’t twisted, overstretched, or in contact with each other, which could alter fibre properties presented to the cells.

Care was taken to ensure that the same density of cells were exposed to the same surface area of suture by slowly dripping the 200μL of cells over the whole visible surface of suture. Once seeded, the plates were placed in a 37°C incubator for 2 hours. Then, crowns were moved to a 24-well plate with 2mL of culture media, so they were suspended above TCP and only the cells attached to the filaments were accounted for. At 24 hours after seeding, samples were removed from the crowns for measuring initial attachment and kept in culture media (without crowns) for prolonged culture. Unless stated otherwise, each experiment was performed with 3 biological repeats and 3 experimental repeats.

Proteomic data analysis

Raw MS data files were analysed using Progenesis QI for Proteomics (Waters) using default settings. MS2 spectra were searched against the human Uniprot reference proteome using MASCOT (Matrix Science) with the following parameters: 10 ppm precursor mass tolerance, 0.05 Da fragment mass tolerance, Oxidation (M) and Deamidation (N,Q) were set as variable modifications, two missed cleavage sites were allowed and peptides were filtered to a 1 % false discovery rate threshold. The mass spectrometry proteomics data have been deposited to ProteomeXchange Consortium via the PRIDE partner repository. Accession number: PXD045312 (username: [reviewer_pxd045312@ebi.ac.uk](mailto:reviewer_pxd045312@ebi.ac.uk) , password: 9oLfIToG).
