# Supplementary material for: A Hybrid Electrospun-Extruded Polydioxanone Suture for Tendon Tissue Regeneration
Source: Tissue Eng Part A. 2024 Mar 15;30(5-6):214–24. doi: 10.1089/ten.tea.2023.0273 (PMC10954604; doi:10.1089/ten.tea.2023.0273)
Supplement: Supplemental data [file Suppl_FigureSA1.docx]

**Supplementary figures**


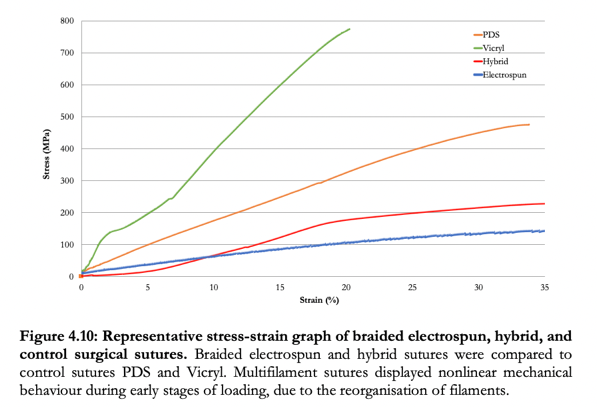


**Figure A1: Representative stress-strain graph of braided electrospun, hybrid, and control surgical sutures.** All sutures failed in the mid-substance region and all multifilament sutures (hybrid, electrospun, Vicryl) showed characteristic toe, linear, and yield regions. The nonlinear mechanical behaviour of the toe region, at 0–5% of the strain, was due to the reorganisation of filaments during early stages of loading and was visible for the multifilament hybrid, electrospun, and Vicryl sutures. The linear region formed at around 5-15% of strain, at which point most fibres were engaged. The sutures yielded from 15% strain with classic post yield softening.
