# Supplementary material for: A Hybrid Electrospun-Extruded Polydioxanone Suture for Tendon Tissue Regeneration
Source: Tissue Eng Part A. 2024 Mar 15;30(5-6):214–24. doi: 10.1089/ten.tea.2023.0273 (PMC10954604; doi:10.1089/ten.tea.2023.0273)
Supplement: Supplemental data [file Suppl_FigureSA2.docx]

**
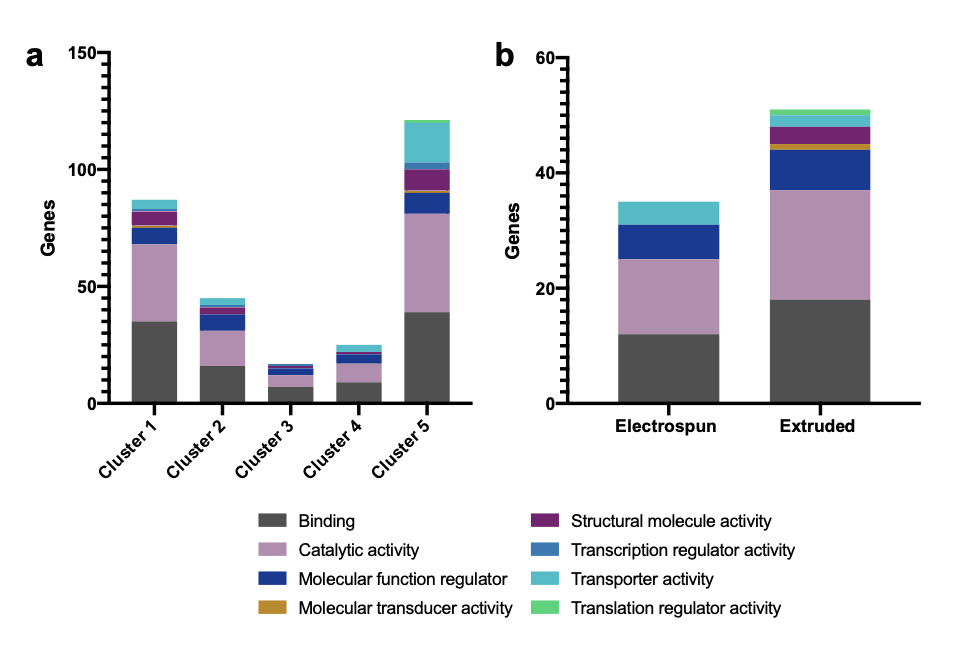
**

**Figure A2: Functional pathways of detected proteins.** (A) Differentially expressed proteins in the coronary of the PDS sutures, electrospun filaments, and melt-extruded filaments formed five main clusters. (B) Differentially expressed proteins in the coronas of electrospun and melt-extruded filaments.
