# Supplementary material for: A Hybrid Electrospun-Extruded Polydioxanone Suture for Tendon Tissue Regeneration
Source: Tissue Eng Part A. 2024 Mar 15;30(5-6):214–24. doi: 10.1089/ten.tea.2023.0273 (PMC10954604; doi:10.1089/ten.tea.2023.0273)
Supplement: Supplemental data [file Suppl_FigureSA3.docx]

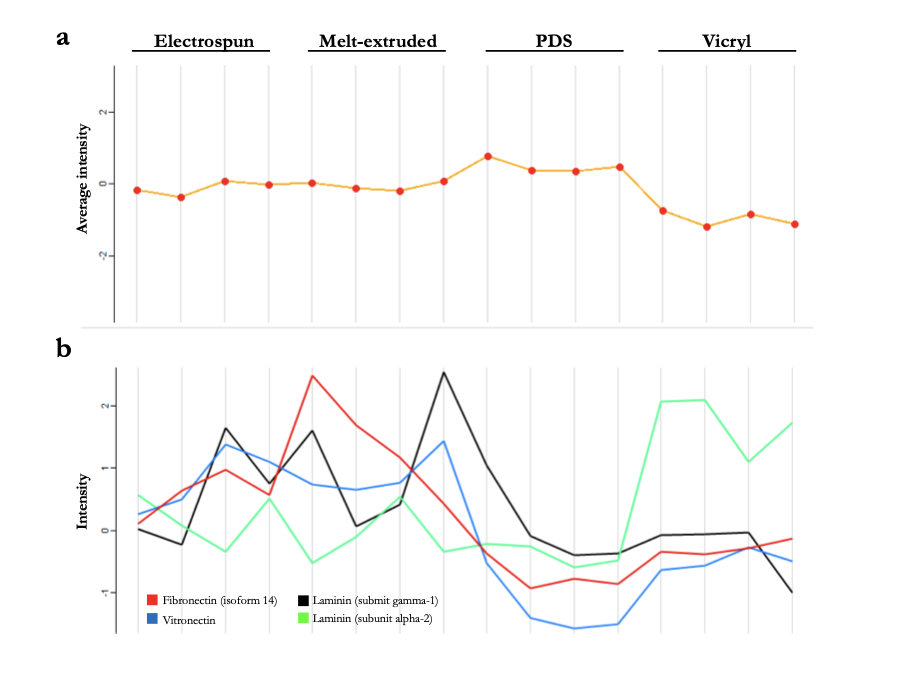


**Figure A3: Differential expression of binding proteins in the coronas of electrospun filaments, melt-extruded filaments, PDS, and Vicryl**. (A) Binding protein abundance calculated by averaging the intensity of all binding proteins for each material. (B) Abundance of specific binding proteins fibronectin, vitronectin, and laminin, that are thought to be important in mediating cell attachment. Each vertical line intersects on sample, i.e. n=4 per material tested.
